# Linking environmental variability to village-scale malaria transmission using a simple immunity model

**DOI:** 10.1186/1756-3305-6-226

**Published:** 2013-08-07

**Authors:** Teresa K Yamana, Arne Bomblies, Ibrahim M Laminou, Jean-Bernard Duchemin, Elfatih A B Eltahir

**Affiliations:** 1 Massachusetts Institute of Technology, Room 48-207, 15 Vassar Street, Cambridge, MA 02139, USA; 2 School of Engineering, University of Vermont, 33 Colchester Ave, Burlington, VT 05405, USA; 3 Centre de Recherche Médicale et Sanitaire, International Network of the Pasteur Institute, Niamey, Niger

**Keywords:** Malaria, Model, Anopheles, Immunity, Africa

## Abstract

**Background:**

Individuals continuously exposed to malaria gradually acquire immunity that protects from severe disease and high levels of parasitization. Acquired immunity has been incorporated into numerous models of malaria transmission of varying levels of complexity (e.g. Bull World Health Organ 50:347, 1974; Am J Trop Med Hyg 75:19, 2006; Math Biosci 90:385–396, 1988). Most such models require prescribing inputs of mosquito biting rates or other entomological or epidemiological information. Here, we present a model with a novel structure that uses environmental controls of mosquito population dynamics to simulate the mosquito biting rates, malaria prevalence as well as variability in protective immunity of the population.

**Methods:**

A simple model of acquired immunity to malaria is presented and tested within the framework of the Hydrology, Entomology and Malaria Transmission Simulator (HYDREMATS), a coupled hydrology and agent-based entomology model. The combined model uses environmental data including rainfall, temperature, and topography to simulate malaria prevalence and level of acquired immunity in the human population. The model is used to demonstrate the effect of acquired immunity on malaria prevalence in two Niger villages that are hydrologically and entomologically very different. Simulations are conducted for the year 2006 and compared to malaria prevalence observations collected from the two villages.

**Results:**

Blood smear samples from children show no clear difference in malaria prevalence between the two villages despite pronounced differences in observed mosquito abundance. The similarity in prevalence is attributed to the moderating effect of acquired immunity, which depends on prior exposure to the parasite through infectious bites - and thus the hydrologically determined mosquito abundance. Modelling the level of acquired immunity can affect village vulnerability to climatic anomalies.

**Conclusions:**

The model presented has a novel structure constituting a mechanistic link between spatial and temporal environmental variability and village-scale malaria transmission. Incorporating acquired immunity into the model has allowed simulation of prevalence in the two villages, and isolation of the effects of acquired immunity in dampening the difference in prevalence between the two villages. Without these effects, the difference in prevalence between the two villages would have been significantly larger in response to the large differences in mosquito populations and the associated biting rates.

## Background

### Acquired immunity to malaria

Malaria transmission in Africa is both widespread and highly variable. In some regions, malaria is endemic with constantly present transmission, whereas other regions remain relatively malaria-free but periodically erupt in epidemics that cause suffering and death, after which the disease recedes again. The regional variability in malaria transmission can be affected by environmental, climatic, and demographic factors, but parasite dynamics are also influenced by the human immune response to the parasite in the bloodstream [1]. Here, we consider the role of acquired immunity to malaria in shaping malaria transmission in two villages in Niger.

Naturally acquired immunity to malaria plays an important role in the transmission of the disease, but in many ways is still poorly understood. Acquired immunity to *Plasmodium falciparum* malaria develops in three stages. The first stage is protection from severe disease, and can develop in as few as one or two infections [2]. The second stage is immunity to the clinical symptoms of malaria, and develops over the first years of childhood. The third stage is a partial protection against parasitization, and develops around adolescence. All three stages of immunity depend on constant transmission. When transmission decreases, immunity weakens [3]. While all three stages of immunity are important to the epidemiology of malaria, for the purposes of modelling disease transmission, we are only concerned with the immunity that protects against parasitization. This immunity potentially affects transmission by reducing the proportion of infectious mosquito bites that result in infection, decreasing the duration of disease and decreasing the infectivity of humans to mosquitoes.

Immune responses to the pre-erythrocytic stages have been shown to be effective at preventing blood-stage infection, forming the basis of the most advanced malaria vaccine to date [4]. Naturally occurring immunity is not believed to confer full protective immunity, and is often neglected in models of disease transmission [5]. However, adults become infected at lower rates than children, implying that immunity does provide a partial protection against infection [6,7].

Immunity decreases parasite levels in the bloodstream, and may lead to shorter duration of infection [8]. In a longitudinal study of recovery rates in Nigeria, the duration of disease in infants (625 days) was 10 times as high as in >44 year olds (52 days), suggesting that acquired immunity increases the rate of disease clearance [6]. Others argue that there is little evidence to support this theory, assuming instead that immunity decreases the length of patent disease, without changing the length of subpatent infection [9].

Gametocyte density has been correlated with the ability to infect mosquitoes, and there is evidence that infectivity to mosquitoes decreases with age [10-12]. However other studies show no correlation, leading to a belief that beyond a bottom threshold of gametocyte density required for transmission of the parasite to mosquitoes, increased densities do not necessarily lead to enhanced transmission [12]. A study of infectivity to mosquitoes in a highly endemic African village found that <5, 5–15, and >15 year old age groups contributed equally to the malaria reservoir, indicating that people continue to be infectious despite having low levels of parasitaemia [13]. It has also been suggested that acquired immunity may decrease the infectivity of gametocytes [12,14].

Beier *et al.*[15] noted a non-linear relationship of malaria prevalence with entomological inoculation rate (EIR: a standard measure of malaria transmission), with steadily rising prevalence levels in the low EIR range (below ~100 infectious bites per person per year), and leveling prevalence at EIR values greater than 200 infectious bites per person per year. In this range, increases in prevalence are low compared to the increase in EIR. At EIR values in the 200–400 range, without immunity, entire populations should be infected with the malaria parasite in a short time. Presumably, the relatively steady 80% prevalence observed in this high EIR range reported by Beier *et al*. [15] can be explained in part by the protective effects of acquired immunity.

Several seemingly paradoxical cases of increased mosquito abundances associated with lower human parasite prevalence have been reported (e.g. [2,16,17]). These results have been attributed to several potential causes, but it is thought that human immunity may play a significant role in explaining these observations. In Mali, Diuk-Wasser *et al*. [16] noted a decrease in malaria prevalence in villages with intensive irrigation and higher mosquito abundance. They suggested intraspecific competition of subadult mosquitoes for limited nutrients as an explanation. The adult mosquitoes would be smaller, shorter-lived, and thus the vectorial capacity would be depressed. However, in addition to the increased nutrient competition resulting from larval crowding, acquired immunity may have played a role in lowering the population prevalence in many of these observations.

### Modeling immunity to malaria

Previous malaria models have incorporated acquired immunity. Dietz *et al*. [18] developed a model that tracks temporal variation in malaria infections and the immunity level of populations in northern Nigeria. That model has a compartmental structure and assumes perfect mixing, and was successfully used for vector control decision making during the Garki project, an extensive malaria control field campaign in the 1970s [18]. Many similar models with a compartmental structure exist with varying levels of complexity and assumptions regarding the mechanisms of immunity, for example Aron *et al*. [19], Yang [20], Filipe *et al*. [21] Chiyaka *et al*. [22], Águas *et al*. [23] and Chitnis *et al*. [24]. Despite substantial differences in model structures, each of the models above have been parameterized in order to matched observed prevalence data.

Several recent models include individual-based humans. Smith and colleagues [25] have developed an extensive stochastic individual-based model driven by the entomological inoculation rate (EIR), the number of infectious bites received by each human per unit time. This model includes modules for pre-erythrocytic immunity that decreases the frequency of infection [5], and parasite regulating immunity at the blood stage that decreases the infectiousness of humans to vectors [26,27]. Similarly, individuals in a model developed by Griffin *et al*. [28] are infected according to the EIR derived from a corresponding compartmental model. This model includes representation for clinical immunity and infection-blocking immunity that developed based on the number of infectious bites received, as well as a parasite regulating immunity that is dependent on the individual’s age. Gu *et al.*[29] developed an agent-based model of humans and female mosquitoes to simulate the transmission of malaria incorporating human immunity for a population on the coast of Kenya. Here, we add to an individual-based model structure by allowing the environmental determinants that influence mosquito breeding and biting activity to vary in high resolution in both space and time, allowing spatio-temporal variability in land use and climate to affect village scale malaria transmission. We present a simple model of immunity, allowing the environmental determinants that are seen to cause spatial and temporal variability in village scale mosquito populations to be represented, and the effects of such variability to be studied in a virtual field environment.

## Methods

### Study location

**Figure 1:**
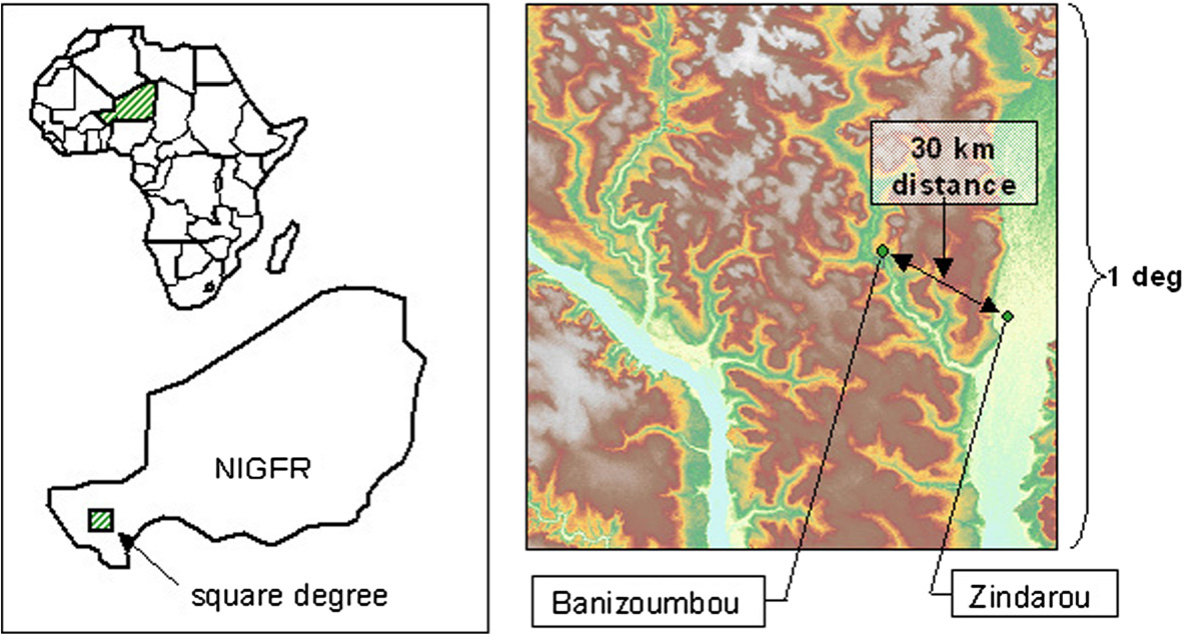
**(From Bomblies *et al*., **[30]**).** Shows the location of the studied villages Banizoumbou ,Zindarou and Niger. The right panel depicts topography within the HAPEX-Sahel square degree, the subject of an intensive international hydrology and climatology research project that took place from 1991 until 1993. The Niger River is seen in the bottom left of the domain, and the “Dallol Bosso” relict river basin is seen on the right. Zindarou’s location within the Dallol Bosso results in the village’s unique hydrology, whereas Banizoumbou has a more arid hydrology that is typical of the Sahel.

**Figure 2:**
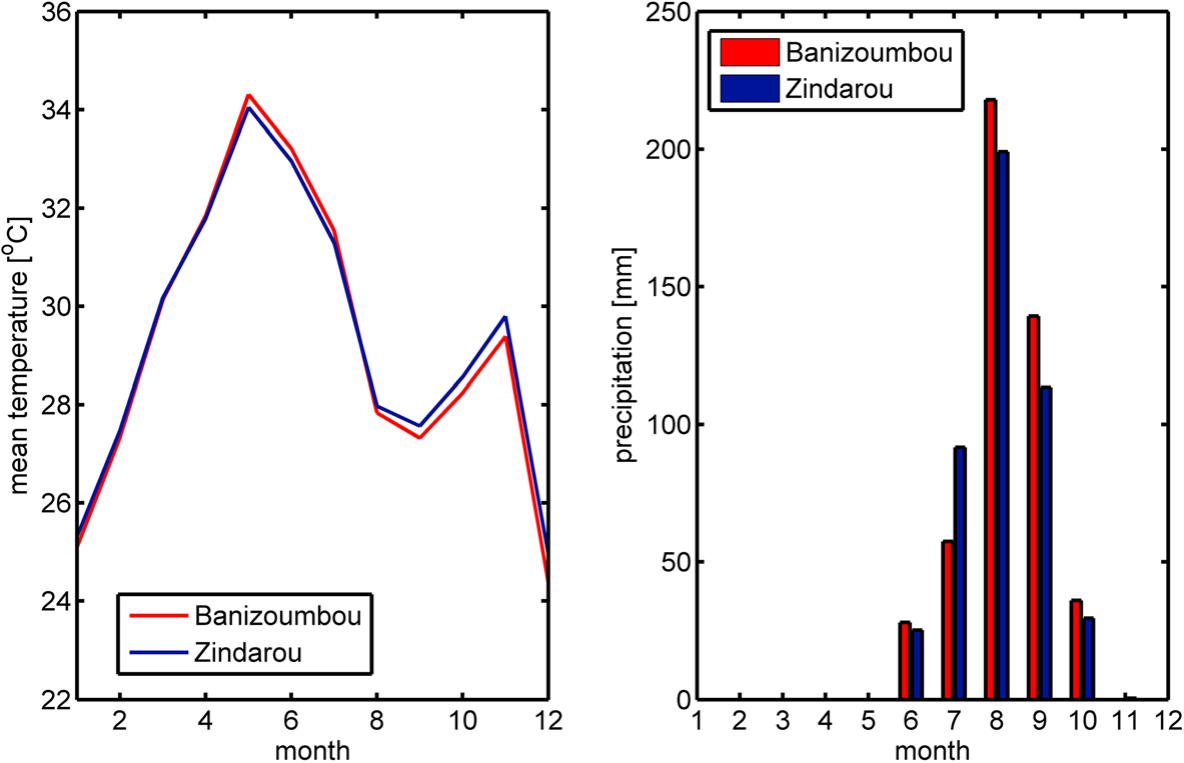
**Temperature and rainfall in Banizoumbou (red) and Zindarou (blue) in 2006.** The figure on the left shows mean temperature, and the figure on the right shows monthly rainfall. This demonstrates that the two villages have very similar climates.

**Figure 3:**
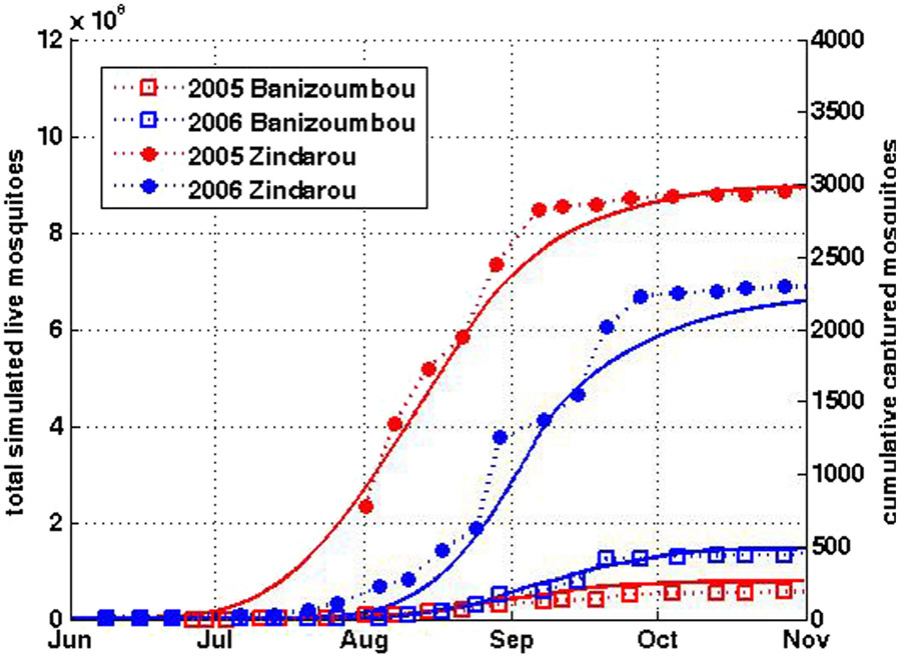
**(From Bomblies *et al*., **[30]**) Modeled and observed Anopheles gambiae mosquito abundance in Banizoumbou and Zindarou.** Mosquito abundance is very different in the two similarly sized villages, because of local hydrological differences. This is evident in the light trap captures (markers with dashed lines) and the simulation results (solid lines).

Located only 30 km apart (see map, Figure 1), Banizoumbou and Zindarou are subject to the same general climate, shown in Figure 2, yet exhibit markedly different mosquito abundance. The difference in mosquito abundance has been shown to be the result of varying hydrological conditions between the two villages [30]. Regional average annual rainfall in this region of Niger is approximately 500mm and occurs exclusively during the summer monsoon (June – September), during which local mosquito populations increase significantly. Banizoumbou is typical of the Sahel, in that it is arid, has deep groundwater and has very little pooled water outside of the summer monsoon season. However, during the summer monsoon season, the arid Sahel landscape is dotted with turbid ephemeral pools that constitute the preferred breeding habitats of *Anopheles gambiae sensu lato* mosquitoes, the dominant malaria vector in the region. Zindarou, on the other hand, has shallow groundwater (depth to water table is ~1 m) because it is located in a relic river channel known as the Dallol Bosso. The Zindarou villagers dig shallow garden wells to access water for their vegetable gardens, which exposes a large water surface area to continuous, perennial mosquito breeding. The pooling of rainfall during the summer rains is also exacerbated by the shallow groundwater, because infiltration causes the shallow groundwater table to rise, creating extensive surface expressions of groundwater [31]. Not surprisingly, this leads to very high mosquito abundance. Figure 3 presents field observations of mosquito abundance in the two villages, showing significantly more mosquitoes in Zindarou than Banizoumbou for both 2005 and 2006.

The hydrological differences between Banizoumbou and Zindarou and the associated differences in mosquito abundance were simulated by Bomblies *et al*. [30] using the highly detailed coupled hydrology and entomology numerical model HYDREMATS (Hydrology, Entomology and Malaria Transmission Simulator) (Figure 3). With meteorological forcing from local meteorological stations, the distributed hydrology model component successfully simulated the differences in hourly surface area of breeding habitat availability, and the agent-based entomology model component coupled to the hydrology model successfully simulated the differences in mosquito populations that were observed using CDC light traps. Despite being very close and subject to nearly the same rainfall and temperature conditions, the two villages have markedly different levels of entomological activity.

In this study, we use HYDREMATS (described below) to investigate the levels of malaria prevalence in the two villages. We do this by extending HYDREMATS to include a representation of transmission of the malaria parasite between humans and mosquitoes. An important aspect to malaria transmission is the semi-protective immunity to disease that is acquired by receiving infectious bites. The resulting partial immunity regulates the density of parasites within the blood, and depends on the intensity of transmission [32]. Therefore, acquired human immunity exerts a moderating effect on malaria transmission, and is expected to be a significant factor in shaping malaria propagation through the human/mosquito transmission cycle by a negative feedback mechanism. It follows that for accurate model representation of linkages between environmental variability and malaria prevalence, effects of human immunity must be considered.

### Description of HYDREMATS model

**Figure 4:**
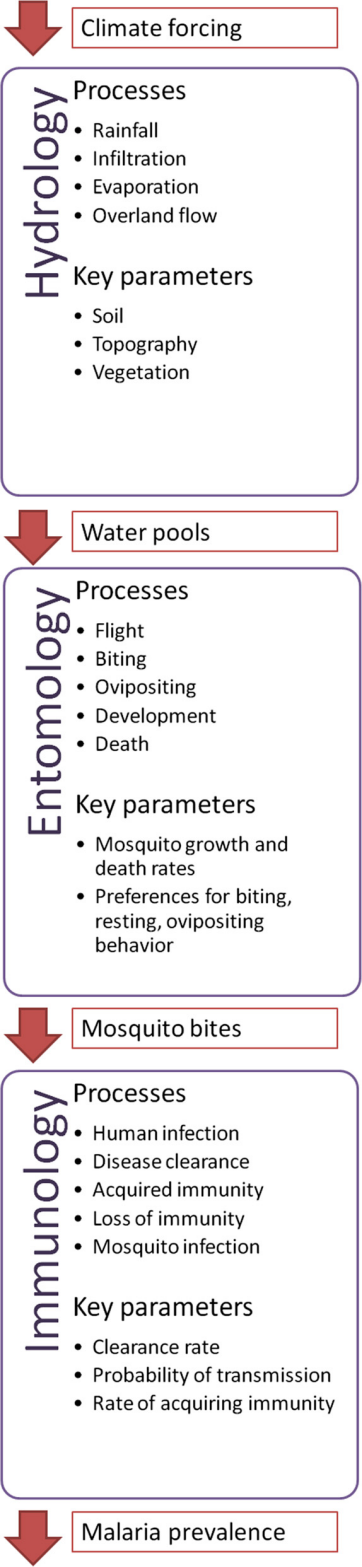
**Schematic of HYDREMATS.** This schematic diagram lists the major processes and key parameters represented by the Hydrology, Entomology and Immunology components of HYDREMATS. The arrows represent information that is passed from one component to the next. Model outputs from each component are spatially and temporally explicit.

The Hydrology, Entomology and Malaria Transmission Simulator (HYDREMATS), was developed by Bomblies *et al.*[33] to simulate the village-scale response of malaria transmission to hydrological and climatological determinants, and has been used in several recent studies in West Africa [30,34-38]. For full details about the development of the hydrology and entomology components of HYDREMATS, and for comparison to field observations of hydrological conditions and mosquito populations, we refer the reader to Bomblies *et al.*[33]. Key features of the model are described in Additional file 1. In this paper, we add an immunology component to the existing hydrology and entomology components of HYDREMATS. A schematic diagram of this combined model is shown in Figure 4. Hydrology is an important proximal determinant that regulates malaria variability at a small scale, because the occurrence and distribution of pooled water often limits the breeding of the *Anopheles* mosquitoes that transmit the malaria parasite. Although such variation also occurs on a large scale, it can be quite pronounced at a very local scale [30,31]. Most models seeking to link environmental and climatic conditions to malaria transmission assume perfect mixing in a compartmental structure. Such a structure does not reflect the importance of small-scale proximal determinants that can influence entomological activity near a village (e.g. [39,40]). High resolution hydrology simulation can represent the important variability in pooled water for mosquito breeding that can regulate village-scale entomological activity [30]. In order to represent this small-scale variation in pooled water, the hydrology component of HYDREMATS uses environmental inputs to mechanistically simulate the runoff of rainfall into water pools and the drying of these pools due to evapotranspiration and infiltration, resulting in the spatial distribution of water depths and temperatures for each 10 meter x 10 meter grid cell, for each 1 hour timestep. These distributions serve as the inputs for the entomology component of the model [33].

The entomology component of HYDREMATS simulates individual mosquitoes and human agents. Human individuals are assumed to be immobile and are assigned to mapped village residences, as malaria transmission in this region occurs primarily at night when humans are indoors [41]. Mosquito agents have a probabilistic response to their environment based on a prescribed set of rules governing dispersal and discrete events including development of larval stages, feeding, egg-laying and death [33].

An agent-based simulation of mosquito population dynamics that maintains spatial structure of the population can link malaria risk with distributed hydrology. Such a model must track the infected status of mosquitoes and individual humans with the transmission moderated by an individual human’s immune status, ultimately linking environmental conditions around an individual village to malaria prevalence within that village. We present such a model in this study, and compare simulated prevalence to field-measured malaria prevalence in Banizoumbou and Zindarou. The resulting combination creates a mechanistic modeling link between environmental variability (e.g. land use patterns, hydrological variability, and climatic variability) and malaria prevalence that maintains the spatial structure of human and mosquito populations and their relationships with the environment. This is the most comprehensive malaria transmission model to our knowledge, mechanistically simulating the hydrological, entomological, and immunological processes involved in linking environmental forcing to indices of malaria transmission (See Figure 4). The Liverpool Malaria Model [42] also simulates processes from environmental inputs through malaria prevalence, but uses a coarser spatial and temporal resolution and compartmental structures for human and mosquito populations, and uses 10-day accumulated rainfall as a proxy for pool availability rather than mechanistically simulating hydrological processes.

### Extension of HYDREMATS model to include immunological processes

The hydrology and entomology components of HYDREMATS were developed by Bomblies *et al*. [33] as an agent-based model in which individual mosquitoes interact with their immediate environment. In this paper, HYDREMATS is extended to simulate the immunological processes of malaria transmission as illustrated by Figure 4. HYDREMATS includes a representation of infected status for both humans and mosquitoes. Infected status is tracked as an attribute of both mosquito and human individuals, and contact between a sporozoite-infected, host-seeking mosquito and a human host can result in an inoculation. Simulated individual mosquitoes are able to acquire parasite infection if they ingest an infectious blood meal, and the infectious stage of the mosquito-borne parasite—the sporozoite—forms after 111 degree-days above 16 degrees C [43]. At this point, the mosquitoes are able to transmit a new infection to the next human bloodmeal host, completing one cycle of transmission. If animal hosts are chosen for a bloodmeal instead of humans, no transmission occurs. Once infected, mosquitoes remain infected for life, and once the parasite has reached the sporozoite stage within the mosquito, it remains in this stage for the duration of the mosquito’s life. In contrast, human individuals clear the parasite at rate *r,* described below.

Since many details of immunity and malaria transmission remain unknown and thus are difficult to parameterize, we present a model with minimal parameters. Whenever possible, parameter values are taken from literature presented in the background section. Where no exact value was given, we assumed parameter values that are consistent with current knowledge. Of course, model results will depend on choice of parameters, and a perfect fit and parameterization is not a goal of this study. Rather, we seek to reproduce general observed trends with a simple model to help understand the effect of immunity in high resolution individual-based models and inform future malaria modeling efforts of such nature.

Human immunity for each human individual is represented in HYDREMATS the index *imm*, which varies from 0 (immunologically naïve) to 1 (fully developed immunity). Each day, the immunity (*imm*) of any human individual that has received at least one infectious bite during the previous 24 hours is raised by parameter *s*, regardless of that human individual’s infected status, up to a maximum of 1. We set parameter *s* to 1/60 per infectious bite, reflecting the slow build up of immunity to parasitaemia through childhood and adolescence [44]. Immunity is lost at a rate of 0.019% per day, corresponding to a half- life of ten years, reflecting the protective effects of immunity on the order of decades in the absence of exposure [32].

Each time a human individual is subjected to an infectious bite, the probability of infection is given by:

$$b={\left(\text{bmin}‒\text{bmax}\right)}^{*}\text{imm}+\text{bmax}$$

where *bmax* and *bmin* are parameters reflecting the probability of infection with no immunity and full immunity, respectively. A uniform random number is generated and compared to *b* to determine if the human individual has acquired the parasite from the infectious bite.

A recent compilation of observed values of *b* gave a range between 0.01 and 0.49 [42]. We reflect this range by setting *bmax*=0.5 and *bmin*=0.05. The non-zero value of bmin allows even fully immune individuals to contribute to the disease reservoir [13].

The duration of a simulated human malaria infection depends on the individual’s immunity level, such that the duration of disease shortens as immunity increases. The duration of each infection is exponentially distributed with rate parameter, *r*, set to

$$r={\left(\text{rmax}‒\text{rmin}\right)}^{*}a+\text{rmin}$$

The value for *rmin* is set to 1/220 days^-1^, which is consistent with the mean duration of infection found in immunologically naïve adults infected with malaria [45]. As we could not find a published estimate for *rmax*, we assume that full immunity doubles the clearance rate and set *rmax* to 1/110 days^-1^. The recovery rate is also affected by superinfection, the state of an individual having two or more concurrent malaria infections, following the assumption made by Macdonald [46] and Dietz *et al*. [18] that multiple infections can occur simultaneously and the duration of each infection is not affected by the presence of other infections. Thus each infection within a human is tracked separately and must be cleared independently at rate *r*.

We did not include an effect of immunity on the probability that a mosquito is infected when biting an infected human, due to the high uncertainty regarding the effect of acquired immunity on human infectivity to mosquitoes. However, this could easily be modified in the model, should more definitive information come to light.

HYDREMATS was modified so that each human in the village population is assigned an age, distributed according to local demographics [47]. The initial immunity level is proportional to an individual’s initial age, reflecting the accumulation of immunity over time. Humans age as the model progresses, and at each time step, they are subjected to a probability of death equivalent to 0.0436/year. In order to maintain a constant population size, a new child is born into the model population each time a human dies.

**Figure 5:**
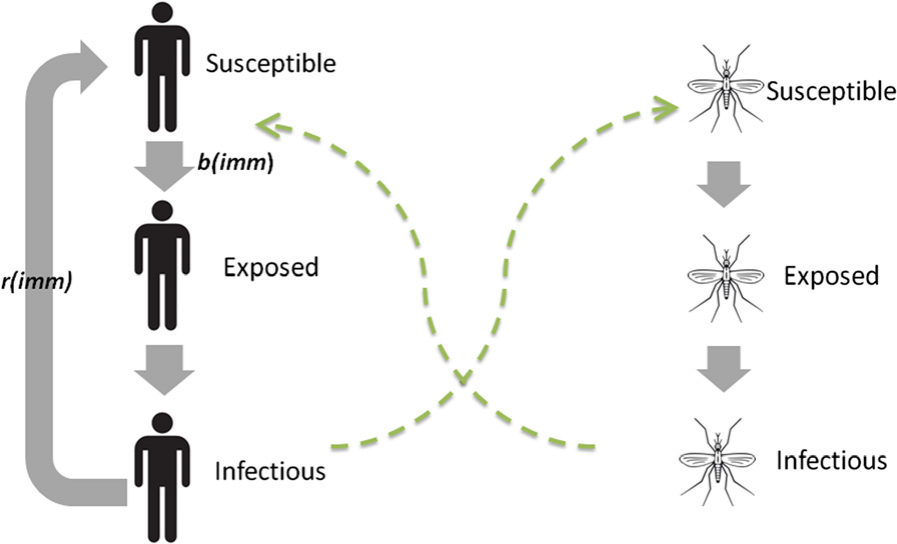
**Schematic of the immunology component of HYDREMATS.** HYDREMATS models individual mosquito human and mosquito agents. The solid arrows represent processes as individual agents become infected, dashed lines indicate the movement of malaria parasite through mosquito bites. Each human agent has an immunity value *imm*, which is a function of the past infectious bites received by that individual. When a human is bitten by an infected mosquito, his probability of infection is *b*, which is a function of *imm.* After a latent period, the exposed human becomes infectious. The human then recovers at a mean rate of *r*, which is also a function of *imm*. A mosquito biting an infectious individual becomes infected with probability *c*. If infected, he goes through a temperature-dependent latent period and then become infectious to subsequent humans.

Figure 5 shows a schematic of the malaria transmission model within HYDREMATS. Solid arrows represent the progress of individual human and mosquito individuals through infectious states, and dashed arrows indicate the transmission of malaria parasites through mosquito bites. The parameters for the immunity model are listed in Table 1. The sensitivity of disease prevalence to parameter values was assessed by perturbing each parameter by 10% and observing the effect on mean annual prevalence after 10 years of simulation in Banizoumbou.

**Table 1:** Parameters for immunology component of HYDREMATS

	**Parameter value**	**Percent change in prevalence parameter value decreased by 10%**
Minimum disease clearance rate parameter	1/220 days	+35%
*rmin*		
Maximum disease clearance rate parameter	1/110 days	+5%
*rmax*		
Rate of acquiring immunity	0.017 per infectious bite	−4%
*s*		
Maximum probability a human is infected when bitten by infectious mosquito	0.5	+1%
*bmax*		
Minimum probability a human is infected when bitten by infectious mosquito	0.05	0%
*bmin*		
Rate of immunity loss	0.019% per day without infectious bite	+6%

With this formulation, malaria prevalence depends on resistance acquired over several years of repeated inoculations within a population. We stress that this is a very simple representation of a very complex and highly developed human immune response to the malaria parasite. Nevertheless, the model representation of immunity captures many of the important aspects regarding the role of immunity in malaria transmission. It allows the effects of immunity on malaria transmission to be incorporated into the model in a flexible and representative manner. This formulation allows simulated prevalence to be compared to observed prevalence while maintaining spatial structure.

### Simulations

To assess the importance of the difference in immunity between the two villages Banizoumbou and Zindarou, we first conduct a simulation for each village where the immunity level for each individual is static, remaining at 0.2 for the duration of the simulation. Climate forcing from 2006, recorded at the each village’s meteorological station, was repeated twenty times in order to achieve a steady state in malaria prevalence and immunity. We then conducted a twenty year simulation in each village using the dynamic immunity model described above, where individuals acquire immunity as they accumulate infectious bites and lose immunity in the absence of inoculations.

### Field observations

Field measurements of malaria prevalence were made in Zindarou and Banizoumbou between December 2005 and February 2007. The populations of Zindarou and Banizoumbou are roughly 500 and 1000, respectively, of which approximately 20% are under the age of 5 [47]. Bimonthly blood samples were taken from approximately 25 children aged one to five years old in each village. The sample size was decided based on financial and practical constraints. Resulting blood smears were analyzed microscopically for parasite presence. Children with observed or reported fever were sent to a local health clinic for treatment in accordance to national malaria treatment guidelines. Ethical clearance was obtained from the National Ethics Committee of Niger.

## Results and discussion

**Figure 6:**
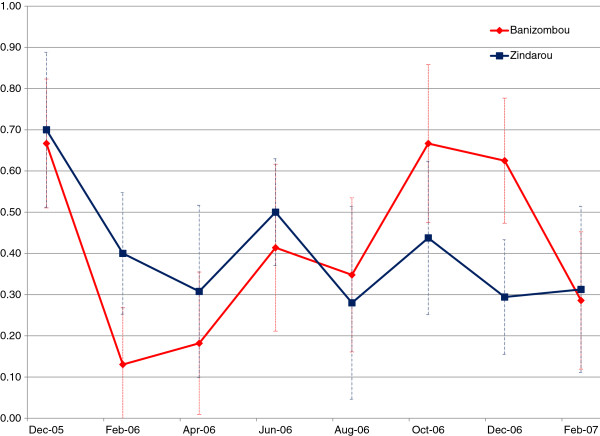
**Observed prevalence in Banizoumbou (red) and Zindarou (blue), for the period December 2005 – February 2007.** Error bars indicate 95% confidence intervals for each estimate.

This study has simulated malaria transmission in two villages, Banizoumbou and Zindarou, Niger, which are subject to nearly identical climatic conditions, but are hydrologically very different. Mosquito captures in both villages during the 2006 rainy season show that abundance in Zindarou is approximately ten times that of Banizoumbou, and other years have shown the same order-of-magnitude difference in light trap captures [33]. However, the prevalence measured in the two villages was not significantly different, despite the order of magnitude difference in mosquito abundance. For the period February 2004 – December 2006, average prevalence was 0.54, 95% CI [0.50, 0.58] in Banizoumbou and 0.56, 95% CI [0.51, 0.61] in Zindarou. Bimonthly prevalence for the year 2006 is shown for both villages in Figure 6. The much higher vector population of Zindarou corresponds to very similar prevalence to that of Banizoumbou. This surprising result suggests that acquired immunity resists the malaria parasite within the human population, and that the high inoculation rate in Zindarou boosts immunity such that prevalence is moderated. Our simple immunity model captured this moderating effect, as shown by comparison of model results to prevalence as determined by blood smears in village children.

**Figure 7:**
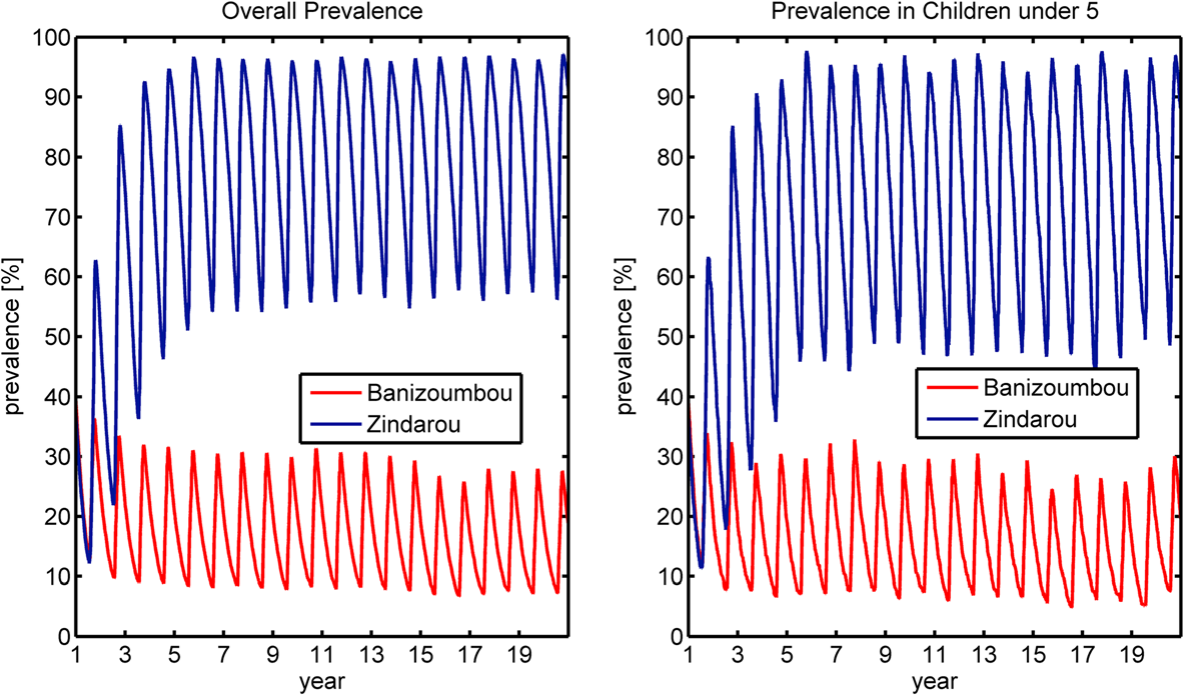
**Simulated malaria prevalence using static immunity model.** Banizoumbou is shown in red, and Zindarou is shown in blue. The left panel shows overall prevalence for all age groups, and the right panel shows prevalence for children under 5. In this simulation, 2006 climate forcing was repeated twenty times. The time step is years, and the cycle is annual. The peaks of each cycle correspond to late August.

We conducted two simulations for each village; one with static immunity where each person’s immunity is set at a constant level of 0.2 throughout the simulation, and one with dynamic immunity where an individual’s immunity level responds to infectious bites. Figure 7 shows the simulated prevalence in the static immunity simulation for the overall population (left panel) as well as for children under five (right panel). As expected, the increased mosquito activity in Zindarou led to higher rates of malaria transmission than in Banizoumbou, and as a result, the prevalence levels in Zindarou are much higher than in Banizoumbou. In both villages, there is a strong seasonal signal in simulated prevalence, consistent with increased biting during the summer monsoon period, which peaks in mid-August in southern Niger. Because this simulation assigns the same immunity level to all human individuals regardless of their age, there is little difference in simulated prevalence between children and adults. The most notable difference is the lower minimum prevalence in children than in adults in both villages. This is the <5 age group and includes the continuous birth of malaria-free humans. Children born during the dry season are likely to remain free of infection until the following transmission season, thus lowering the average prevalence of this age group.

**Figure 8:**
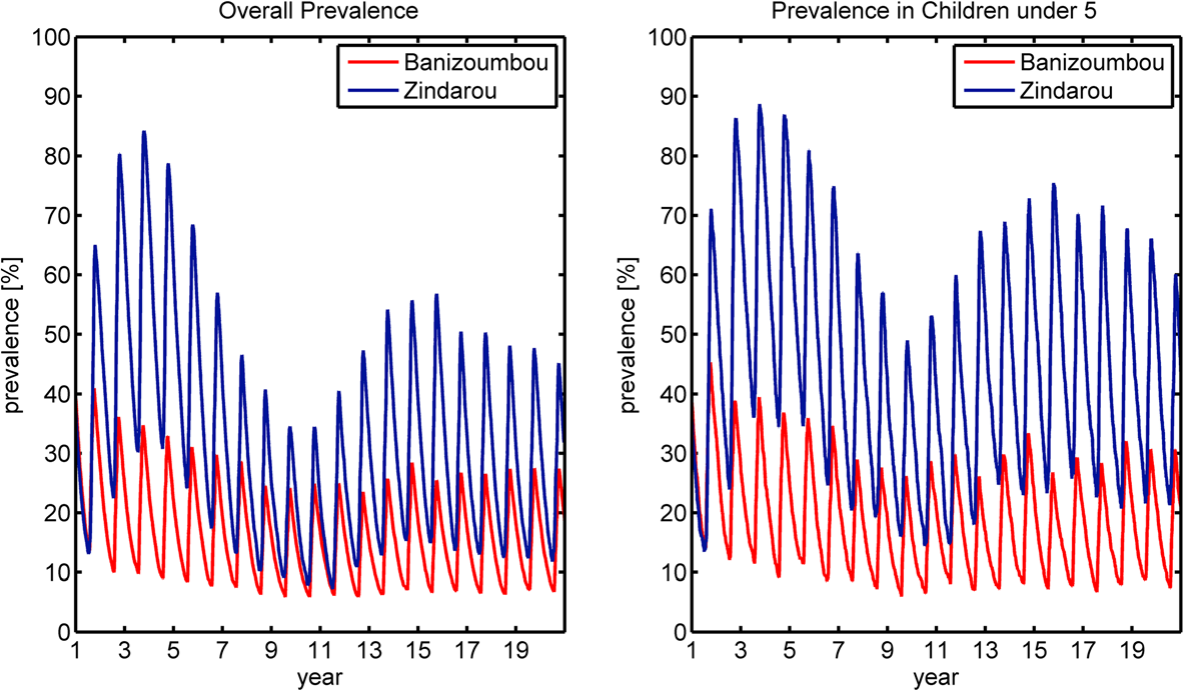
**Simulated malaria prevalence using dynamic static immunity model.** Banizoumbou is shown in red, and Zindarou is shown in blue. The left panel shows overall prevalence for all age groups, and the right panel shows prevalence for children under 5. In this simulation, 2006 climate forcing was repeated twenty times. The time step is years, and the cycle is annual. The peaks of each cycle correspond to late August.

**Figure 9:**
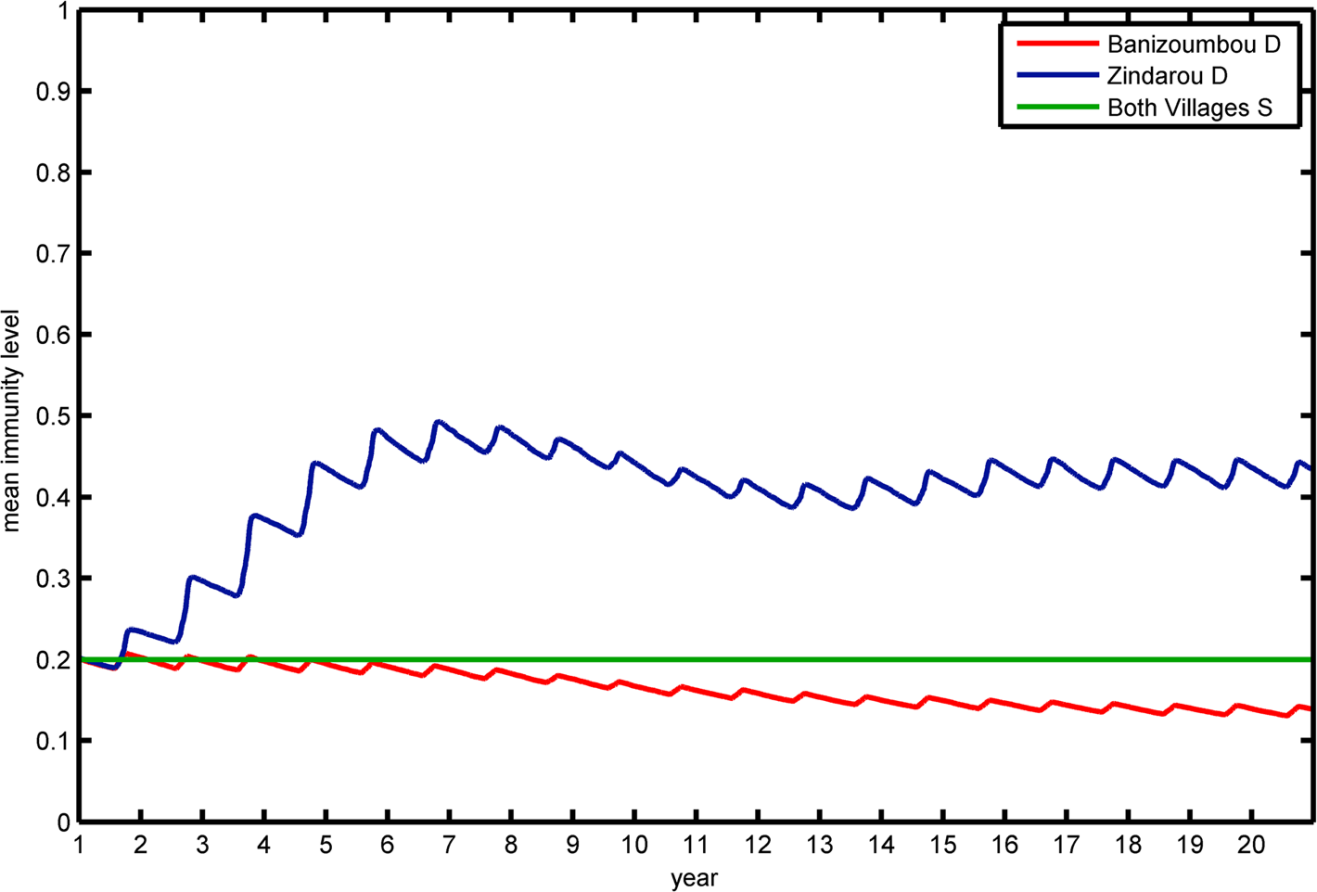
**Simulated mean immunity level using the dynamic immunity model in Banizoumbou (red) and Zindarou (blue).** In the simulations using static immunity, the immunity in both villages remained at 0.2 for the duration of the simulation.

In contrast to the static immunity simulations, the dynamic immunity model results in higher immunity levels in Zindarou than in Banizoumbou as a result of the greater mosquito population in Zindarou. The resulting simulated malaria prevalence for each village is shown in the left panel of Figure 8. Here, the difference in prevalence between the two villages is dramatically reduced. Banizoumbou has relatively low prevalence for the duration of the simulation. Zindarou initially has higher levels of prevalence, until the increased transmission raises population immunity and prevalence rates begin to decrease. The mean immunity levels in the two villages are shown in Figure 9. Mean immunity in both villages begins at 0.2, as individuals are given an initial value of *a* consistent with their age. In Banizoumbou, the mean immunity level decreases slightly to an equilibrium value between 0.16 and 0.18, while in Zindarou the level increases in response to greater numbers of infectious bites.

We can also compare the malaria prevalence in children under five years old, shown in the right panel of Figure 9. In this age group, we see greater differences in prevalence between the two villages. In Banizoumbou malaria prevalence in the <5 year old group is very similar to prevalence in adults, as even adults do not have very high levels of immunity. In contrast, the higher inoculation rate in Zindarou leads to higher immunity in adults, so the prevalence in <5 year olds is higher than the general population.

The simulated prevalence levels in Zindarou were between 20% and 70%, which is consistent with the field observations of prevalence. However, the simulations in Banizoumbou underestimated prevalence, ranging from 8% in the dry season to 30% at the peak of the malaria season. As a result, our simulations show higher prevalence in Zindarou than in Banizoumbou, while field observations do now show a significant difference in prevalence between the two villages. Despite not perfectly replicating the observed prevalence in the two villages, our simulations support the hypothesis that acquired immunity to malaria dampens the difference in prevalence between the two villages that may have been expected given the difference in mosquito populations. In the static immunity simulations, the mean annual prevalence was 59 percentage points higher in Zindarou than in Banizoumbou. In the dynamic immunity simulation, the difference in prevalence between the two villages drops to 22 percentage points.

The improved ability of the model to simulate observed prevalence compared to prevalence without immunity underscores the importance of the negative feedback associated with immunity in the linkage of environmental variability and malaria. However, there are several factors that may be contributing to the difference between simulated and observed prevalence. One possible source of error is the parameterization of the entomology model. While the model properly reproduces relative differences observed in mosquitoes captured by light traps in each village [30], it is not possible to compare the number of simulated mosquitoes to total mosquito population in the village. It is possible that both villages have more mosquitoes than are simulated under current parameterization. Another possible source of error is the parameterization of the immunity model. In our sensitivity analysis, we found that the model was most sensitive to disease clearance rate in people with no immunity (*rmin*). A longer mean duration of infection would lead to higher prevalence in both villages, especially in Banizoumbou, where the lower immunity rates mean that the recovery rate is closer to *rmin* than in Zindarou, where higher immunity rates increase the recovery rate. While the parameter *rmin* was set to 1/220 day^-1^ based on data from immunologically naïve adults [45], it is certainly possible that the clearance rate is different in the study population. For example, one study estimated the mean duration of infection in children 1–4 years old to be 625 days [6]. A third possible source of error is the sampling of village children for the prevalence data. Children testing positive for malaria at each bi-weekly sampling were treated with anti-malarial drugs. Because there were twice as many children in Banizoumbou compared to Zindarou, it was more likely for a child selected for testing in Zindarou to have been tested and treated in the past. Also, the relatively small sample size leads to a wide variance in prevalence estimates.

**Figure 10:**
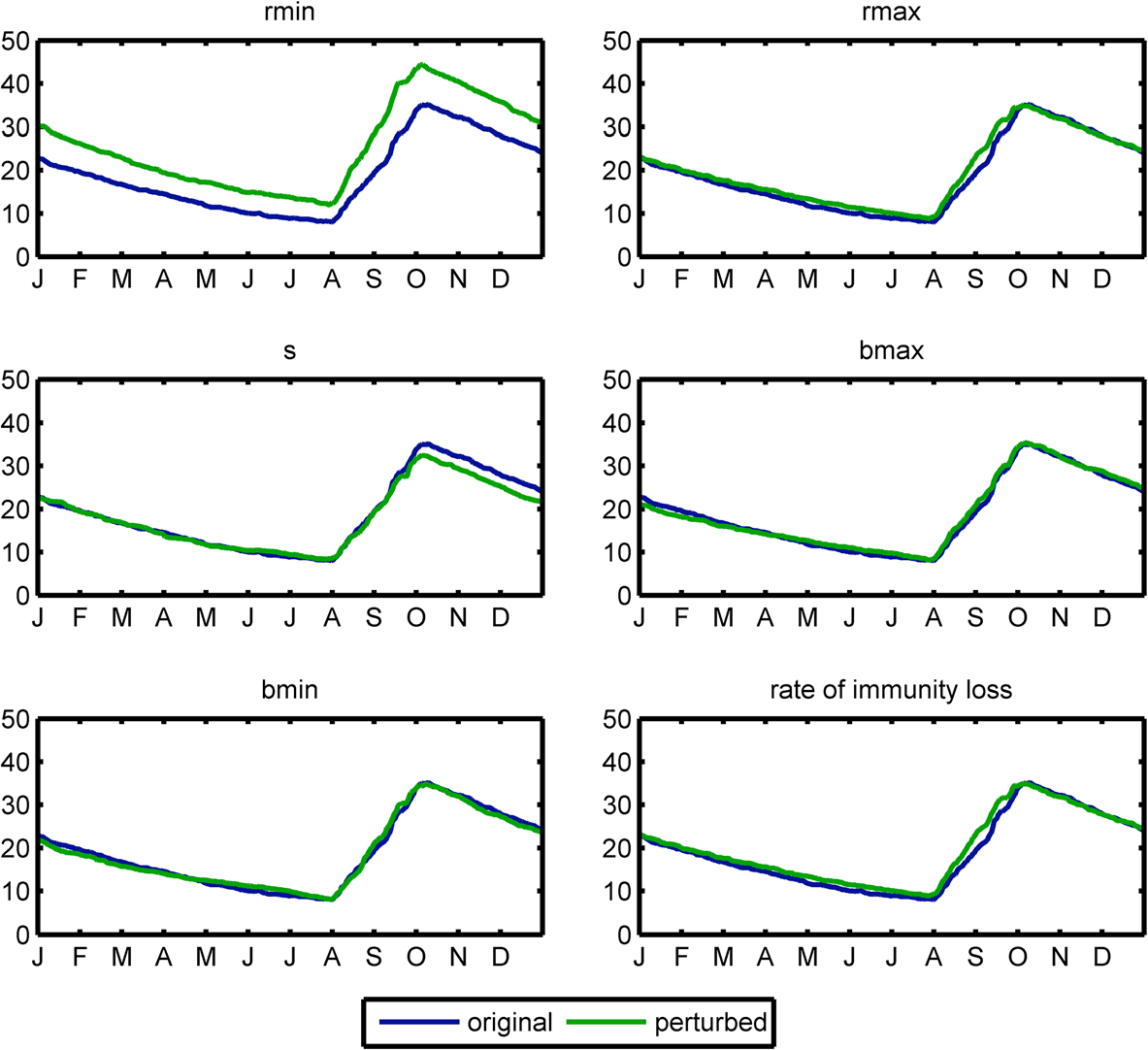
**Sensitivity of model results to parameter values.** Each parameter was decreased by 10%. Prevalence in Banizoumbou after 10 years of simulation under original parameterization (blue) and perturbed parameter (green) are shown.

The model sensitivity analysis indicated greatest sensitivity to the value of *rmin*, with a 35% increase in prevalence when the minimum recovery rate was decreased by 10%. The duration of infection is important in sustaining malaria transmission in areas with low and highly seasonal transmission [29]. Long infections carry the parasite over from one transmission season to the next. The sensitivity analysis showed low sensitivity to other parameter values, with no perturbation leading to more than 6% change in mean prevalence. Results of the sensitivity analysis are shown in Figure 10 and Table 1.

The combined model we presented here, which includes a simple representation of acquired immunity, completes a mechanistic modeling linkage between hydrological variability and village-scale malaria dynamics. While malaria models incorporating immunity are not new, the presented model has a novel structure that allows the spatially- and temporally-varying environmental controls of mosquito population dynamics to determine prevalence as well as variability in protective immunity of the population. This is the only model to our knowledge that provides an explicit link from environmental inputs to malaria prevalence through modelling hydrological, entomological and immunological processes.

While the simulation of overall village prevalence shown in this study could have been achieved using one of the many existing compartmental models of malaria transmission [18-24] driven by the time-series of mosquito biting rates simulated by HYDREMATS, we developed an individual-based model in order to provide a framework for using spatially-explicit individual based models to link environmental variability to malaria transmission in human populations, with the ultimate goal of simulating the impact of environmental changes (changes in regional climate, climate variability and land use) on malaria transmission in human populations at the village scale. The spatially explicit structure of this model allows the spatial relationships between developmental habitat and human population to be maintained. Such a structure is important when simulating the individual mosquitoes that make up a population and ultimately deliver the inoculations leading to malaria prevalence and immunity, because their behavioral decisions depend on their immediate environment and thus distributed land use and hydrology characteristics. Pooled water some distance from a village will host far fewer subadult mosquitoes than pooled water close to the village or within the village itself because the proximity of pools to human hosts makes them much more easily accessible to ovipositing female mosquitoes and hence more likely to be utilized for breeding. In short, a person’s location within a village can influence malaria risk, as risk is heterogeneous [48,49]. Flight distances of mosquitoes from developmental habitat to human blood meal hosts also affects the chances of encounters leading to inoculations, which the presented spatial model represents. The number of inoculations thus depends on spatial relationships of hosts and habitat, and in turn affects the immunity and prevalence levels. The model presented represents a mechanistic linkage between spatial and temporal environmental variability and malaria prevalence in human populations. By tracking the past exposure of each individual human, the individual-based approach provides a more realistic representation of the processes of malaria transmission than compartmental models.

The moderating effect of immunity on malaria prevalence has been shown by others. Dietz *et al.*[18] compared two Nigerian villages and found that despite significant differences in vectorial capacity there were only modest differences in malaria prevalence. This information was used in developing the immunity and superinfection aspects of their malaria transmission model. An entomological and parasitological survey in The Gambia found a negative correlation between vector abundance and malaria prevalence, suggesting substantial differences in immunity between neighboring villages [50]. Macdonald [51] also emphasized the importance of acquired immunity in regulating malaria transmission in the context of field observations and modeling results, and cautioned against incomplete anti-malaria interventions that weaken a population’s immunity, an effect that is now called rebound malaria and has been observed following interventions [52-54]. Our results are consistent with observations that large differences in EIR do not necessarily lead to changes in prevalence [15].

Simulated malaria prevalence in the human population is useful for comparison to field observations; however, a more useful metric for the vulnerability of a village population to malaria epidemics stemming from climate variability, is the immunity level. Zindarou has higher immunity levels than Banizoumbou due to the large number of inoculations resulting from higher vectorial capacity. Anomalously wet climate conditions would boost mosquito populations, and would plague each with higher-than-normal mosquito abundance. Zindarou, with a higher immunity level, would be able to withstand the increased force of infection more than Banizoumbou. The greater immunological defense in Zindarou protects the human population more than in Banizoumbou. Climatic variability brings about differences in vectorial capacity, and the resulting changes in rates of malaria inoculation boost the immune response, which wanes slowly. Repeated years of high vectorial capacity bring about high population immunity, and several years without high levels of transmission can cause immunity levels to wane. Such conditions can make populations very susceptible to devastating malaria epidemics when conditions shift back to promote more intensive biting pressure.

## Conclusion

A simple representation of malaria transmission and the acquired immunity to malaria was developed and embedded in an agent-based model of host-vector-parasite interactions surrounding two villages in Niger, allowing us to simulate prevalence in the two villages, and to observe the effects of acquired immunity. Although the simulated prevalence does not exactly match observations, it does show how acquired immunity dampens the effect of increased biting. Without the effects of immunity, Zindarou would have much higher prevalence than Banizoumbou. However, when we include the effect of immunity, prevalence in Zindarou significantly decreases and approaches Banizoumbou levels. We attribute the lack of discernible difference in measured prevalence between the villages despite pronounced differences in vector abundance to the moderating effect of acquired immunity. Although model and field data from this study suggest that village scale malaria prevalence is largely independent of hydrologic conditions, according to the model the danger to the community depends on the level of acquired immunity, which in turn depends on hydrologically driven mosquito abundance over previous years. Greater acquired immunity in wet Zindarou resulting from more inoculations than in dry Banizoumbou renders the villagers more resilient and less vulnerable to malaria epidemics associated with climate anomalies (e.g. unusually wet years) than their counterparts in Banizoumbou.

## Competing interests

The authors declare that they have no competing interests.

## Authors’ contributions

TKY and AB conducted the modelling study and drafted the manuscript. EABE supervised the study, provided advice for study design and implementation, and participated in writing the final version of the manuscript. JBD and IML provided malaria prevalence data. All authors read and approved the final manuscript.

## Supplementary Material

Additional file 1 Additional details of the HYDREMATS model. Click here for file
