# Supplementary material for: Linking environmental variability to village-scale malaria transmission using a simple immunity model
Source: Parasit Vectors. 2013 Aug 7;6:226. doi: 10.1186/1756-3305-6-226 (PMC3750354; doi:10.1186/1756-3305-6-226)
Supplement: Additional file 1 — Additional details of the HYDREMATS model. [file 1756-3305-6-226-S1.pdf]

# ADDITIONAL DETAILS OF THE HYDREMATS MODEL

The Hydrology, Entomology and Malaria Transmission Simulator (HYDREMATS), was developed by Bomblies et al. [1] to simulate the village-scale response of malaria transmission to hydrological and climatological determinants, and has been used in several recent studies in West Africa [2-7]. For full details about HYDREMATS, and for comparison to field observations of hydrological conditions and mosquito populations, we refer the reader to Bomblies et al. [1]. Here, we repeat the key features of the model in order to orient the reader.

## HYDROLOGY COMPONENT

Because spatial distributions of pools relative to human habitation are so important and can play a large role in transmission intensity [8, 9] a gridded region surrounding human habitation forms the HYDREMATS model domain and individual pool locations are predicted using fine-scale topography as hydrology model input. Simulated pools in topographic depressions host sub-adult mosquitoes, which emerge as individual "agents" if their host pool persists long enough, and are free to interact with their simulated environment within the model domain based on a set of pre-assigned rules and attributes [1].

Pool persistence is a key mosquito population control. To inform the presented model, many field observations were made in a region of southwestern Niger considered representative of the Sahel. In this field environment, rainfed pools and their entomological activity were monitored regularly. Pool desiccation kills all aquatic stage mosquitoes [10]. If they manage to emerge before desiccation, however, mosquitoes will plague humans living nearby. Interaction of simulated mosquito "agents" with immobile human agents facilitates virtual malaria transmission [1].

### *OVERLAND FLOW*

Pool formation is simulated by distributed flow routing. A finite difference solution of a diffusion wave approximation to the St. Venant equations determines routed and pooled water for each time step. Run-on onto down-gradient grid cells combines with available precipitation for the next iteration of the unsaturated zone model. In this manner, shallow flow over a spatially variable infiltrating surface is simulated. Flow velocity is represented by Manning's equation with distributed roughness parameter n. The formulation follows that of Lal [11]. The continuity equation for shallow flow is:

$$\frac{\partial h}{\partial t}+\frac{\partial (hu)}{\partial x}+\frac{\partial (hv)}{\partial y}-P+I+ET=0 \tag{1}$$

where u and v are the flow velocities in the x and y directions, respectively, h is the water depth, P is precipitation, I is infiltration, and ET is evapotranspiration [1].

The momentum equations for the x and y directions are:

$$\frac{\partial (hu)}{\partial t}+\frac{\partial (u^2 h)}{\partial x}+\frac{\partial (uvh)}{\partial y}+hg\frac{\partial (h+z)}{\partial x}+ghS_{fx}=0 \tag{2}$$

$$\frac{\partial (hv)}{\partial t}+\frac{\partial (v^2 h)}{\partial y}+\frac{\partial (uvh)}{\partial x}+hg\frac{\partial (h+z)}{\partial y}+ghS_{fy}=0 \tag{3}$$

where g is the gravitational acceleration, and $S_{fx}$ and $S_{fy}$ are the friction slopes in the x and y directions, respectively. For the diffusion wave approximation, we neglect the first three terms which represent inertial effects. We make the replacement H = h + z for water level above a datum. Equations 2 and 3 then reduce to

$$\frac{\partial H}{\partial x}=-S_{fx} \tag{4}$$

$$\frac{\partial H}{\partial y}=-S_{fy} \tag{5}$$

Manning's equation relates flow velocity to friction slope and flow depth. For the x-direction:

$$u=\frac{1}{n}h^{\frac{2}{3}}S_{fx}^{\frac{1}{2}} \tag{6}$$

where *n* is the Manning's roughness coefficient which determines resistance to overland flow. The y direction velocity is formulated similarly. Following Lal [11], we reformulate equation 6 in terms of H and n:

$$u=-\frac{h^{\frac{2}{3}}}{n\sqrt{S_{fx}}}\frac{\partial H}{\partial x}=-\frac{K}{h}\frac{\partial H}{\partial x} \tag{7}$$

$$v = -\frac{h^{\frac{2}{3}}}{n\sqrt{S_{fy}}}\frac{\partial H}{\partial y} = -\frac{K}{h}\frac{\partial H}{\partial y} \quad (8)$$

with $K = \frac{h^{\frac{5}{3}}}{n\sqrt{S_f}}$

Equations 1, 4, 5, 7 and 8 are then solved using the alternating-direction implicit (ADI) method, as described in Lal [11].

Topography at very high resolution is a critical parameter for overland flow simulation and prediction of pool formation. Topography determines the cell-to-cell bed slope, which is then used to determine intercell flow potentials [11]. The model uses a digital elevation model (DEM) which was derived from a combination of a ground topographic survey and Envisat synthetic aperture radar data [12]. In addition to topography, Manning's n in equation 6 strongly controls the timing and volume of hydrographs entering topographic depressions. This roughness parameter depends on the vegetation cover and soil type at the grid cell, and influences overland flow velocities [1].

### *LAND SURFACE MODEL*

The model presented borrows heavily from the land surface scheme LSX of Pollard and Thomson [13]. The model simulates six soil layers and two vegetation layers for a detailed representation of hydrologic processes in the vertical column. LSX simulates momentum, energy, and water fluxes between the vegetation layers, soil, and the atmosphere. Vegetation type and soil type strongly influence soil moisture profile simulation, and spatially variable soil and vegetation properties are used to assign roughness in the runoff routing model.

Vertical soil layer thicknesses are assigned to allow simulation of a low-permeability structural crust commonly observed at the land surface in bare soil and sparsely vegetated areas of the Sahel [14]. Precipitation at each grid cell is partitioned between runoff and infiltration, based on hortonian runoff processes. The resulting infiltration flux is redistributed in the unsaturated zone with a Richard's equation solver, with soil hydraulic parameters assigned for each layer and grid cell. The Richards equation governs vertical water movement through the unsaturated zone, for which the model uses an implicit solver. The Richards equation is presented in equation 9:

$$\frac{\partial \theta(z,t)}{\partial t} = \frac{\partial}{\partial z}\left[K_u(\theta)\frac{\partial \phi(\theta,z)}{\partial z} + K_u(\theta)\right] \quad (9)$$

where $\theta$ = soil moisture [$cm^3$ $cm^{-3}$]

$K_u(\theta)$ = unsaturated hydraulic conductivity [m $sec^{-1}$]

$\phi$ ($\theta$,z)= head value [m]

z = elevation [m]

Groundwater representation is similar to the lumped aquifer model of Yeh and Eltahir [15]. The regional unconfined aquifer is represented using a lumped model in which groundwater table fluctuations are simulated. The depth to the water table varies from cell to cell and is a function of topography. This addition to the model allows areas of groundwater penetration of the surface and the resulting extended pool persistence to be predicted [1].

### *MODEL INPUTS*

Necessary model inputs come from a variety of sources. The climate data for model forcing can come from meteorological stations in the field, and/or from regional climate model simulations. Meteorologic variable inputs for the hydrology model are temperature and humidity, wind speed and direction, incoming solar radiation, and precipitation. These six variables can be assumed spatially invariant over the model domain, or can be represented as distributed rasters, based on either multiple measurements or assumptions, to account for the existence of mosquito microhabitats. Table 1 summarizes all hydrology model inputs.

Table 1. Hydrology model inputs. Reprinted from “Hydrology of malaria: Model development and application to a Sahelian village,” by Bomblies A, Duchemin JB, Eltahir EAB. 2008. Water Resour Res 44.

| variable | Type | Remarks |
|---|---|---|
| vegetation | distributed | supervised classification of multispectral satellite image (eg Landsat) |
| roughness | distributed | Assigned based on vegetation classification |
| soil type | distributed | supervised classification, and knowledge of local soil compositions |
| topography | distributed | Synthetic aperture radar products (eg Radarsat), or other suitable DEM source |
| precipitation | lumped | from meteorological station or climate model output |
| temperature | lumped | from meteorological station or climate model output |
| humidity | lumped | from meteorological station or climate model output |
| wind speed | lumped | from meteorological station or climate model output |
| wind dir | lumped | from meteorological station or climate model output |
| SW radiation | lumped | from meteorological station or climate model output |
| grid resolution | user-defined | flexible telescopic mesh refinement grid layout to accommodate area of interest |
| time step | user-defined | different time steps as input for overland flow and unsaturated zone models |

## Entomology component

Malaria response to environmental determinants is simulated using individual mosquito and human "agents". Mobile individual mosquito agents behave probabilistically according to a prescribed set of rules governing dispersal and discrete events (e.g. bloodmeals, egg-laying, etc), in response to their immediate environment. This formulation allows population behavior of both mosquitoes and malaria parasites to emerge based on the individuals' actions. Characteristics of each mosquito such as location and gonotrophic or infective status are tracked through time. Interaction with the human population, acquisition of infection, intrinsic and extrinsic incubation periods (parasite development time in humans and mosquitoes, respectively), and infectious bites upon subsequent contact with humans are all simulated in the described manner [1].

### *Model inputs*

Air temperature, water temperature, humidity, wind speed, wind direction, and distributed water depths are the primary inputs for the entomology model. Water depth and temperature for each grid cell are predicted by the hydrology model, and the remaining four can be either field measured or supplied by climate models [1].

### *Aquatic stage simulation*

Aquatic stage, or subadult, mosquitoes advance through several stages between eggs and adult mosquitoes. As shown in Figure 4, eggs hatch to become L1, or first stage larvae. They then advance through three more larval stages (instars) as they grow and mature, to finally pupate. Pupae do not feed. They remain in this state for approximately two days before emerging as adult mosquitoes [1].

Simulation of aquatic stage development relies on a compartmental structure model for each grid cell in which the hydrology model assigns a pool. As long as the pool persists in the simulation, the aquatic stage model will continue to advance. In pools predicted by the hydrology model to disappear, any simulated aquatic stages will be killed in the simulation, as is expected of naturally occurring *An. gambiae* larvae and pupae upon desiccation [10]. An aquatic-stage model structure is embedded within each model grid cell containing water. This model describes the water temperature-dependent stage progression rates of eggs, larvae, pupae, and emerging adults. Only integer abundances are advanced from a previous stage to the next. Progression from eggs to larvae to pupae to adults is calculated using Depinay's temperature-dependent model [16]:

$$d_k = r(T_k) \cdot \Delta t_k \qquad (10)$$

where d is the fraction of individuals in a certain stage progressed to the next stage, $T_k$ is the temperature (K) over time interval k, $\Delta t_k$ is the time step at interval k, and $r(T_k)$ is the temperature-dependent development rate, given by Depinay et al. [16] as a function of water temperature and biochemical parameters specific to each subadult stage. For details of the temperature dependence, the reader is referred to Depinay et al. [16]. For all stages, predation and natural mortality are model parameters [1].

Following Depinay et al. [16], we limit pool biomass using an intraspecific competition coefficient defined as:

$$C = \left( \frac{e - w}{e} \right) \tag{11}$$

where w is the sum of total larval biomass in the pool grid cell, and e is the ecological carrying capacity [mg biomass $m^{-2}$]. Ecological carrying capacity is an assigned model parameter and is assumed to be time-invariant [1].

Several other factors influence larvae. Pool water temperatures in excess of 40 degrees result in death of larvae [16]. In addition, we assume that oviposition does not occur in pools deeper than a threshold depth [17]. This is consistent with our own observations that wave action (which generally occurs in deeper, larger, unvegetated pools) seems to deter larvae, either by wave action drowning them or by waves discouraging oviposition. Also, deep water in the center of large pools appears to contain virtually no larvae. In the hydrology simulation, shrinking pools will regularly dry out grid cells at the pool edges as the receding water line causes a retreat of the pool boundaries. As soon as one pool grid cell is predicted to become dry, all subadult mosquitoes are simply moved into the adjacent cells, concentrating larvae and pupae into remaining pool cells [1].

### *ADULT STAGE SIMULATION*

After emergence from the pools, adult mosquitoes are tracked through space and time using an individual-based approach, in contrast to the compartmental structure of the aquatic stage simulation. At each time step in the model, after the aquatic routine has been stepped to simulate newly emerging adult mosquitoes in the simulation, the mosquito matrix is updated. Elapsed times since significant events are updated, and X and Y position and behavior of the mosquito are updated based on radial random walk motion, corrected for wind displacement and including representation of $CO_2$ plumes as a host-seeking cue, as described below. We assume the following sequence of behavioral events: host-seeking, biting, resting, oviposition, and again host-seeking to repeat the cycle until the mosquito dies. Figure S1 shows adult

stage model flow. The model cycles through each individual, assesses the mosquito's gonotrophic and infectious states and updates the mosquito attributes for infection and bloodmeals based on interaction with the environment or human agents at that individual mosquito's location. Each time step only allows events which occur within the flight range of one time step. The model simulates the diurnal cycle, and allows mosquito activity only during the evening and nighttime hours. Anophelines are assumed to rest during the day, either in houses or in nearby vegetation [1].

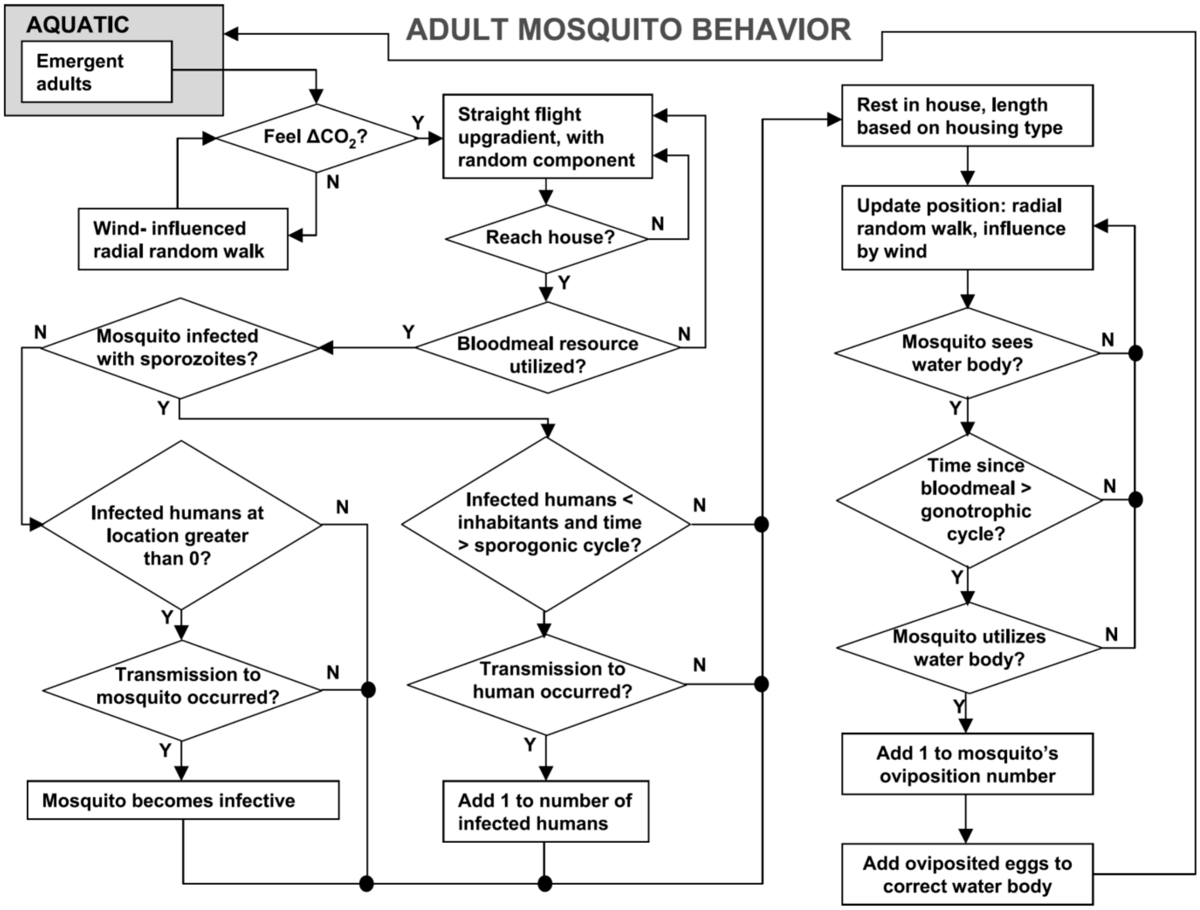


Figure S1 Adult mosquito simulation flow. During each timestep, the model updates each individual mosquito as she progresses through her life cycle. Mosquito attributes are updated as they interact with the environment and human agents. Reprinted from "Hydrology of malaria: Model development and application to a Sahelian village," by Bomblies A, Duchemin JB, Eltahir EAB. 2008. Water Resour Res 44.

The model incorporates a daily survivability based on daily average temperatures. Above a 42 ˚C daily average temperature threshold, anophelines cannot survive. [18, 19]. The survivability dependence on daily average temperature follows the model developed by Martens [19]:

$$p = \exp\left(\frac{-1}{-4.4 + 1.31T_d - 0.03T_d^{\,2}}\right) \qquad (12)$$

where p is the daily survivability probability of each mosquito and $T_d$ is the average temperature of the previous 24 hours [1].

### *EGG DEVELOPMENT AND EXTRINSIC INCUBATION PERIOD*

Egg development within the mosquito follows the temperature-dependent model of Depinay et al. [16] as shown in equation 14. For details of the temperature-dependent development rate of eggs within the mosquito, the reader is referred to Depinay et al [16]. Ambient temperature at the mosquito's location regulates this development rate. If the mosquito has finished the full gonotrophic (egg development) cycle, and it encounters a suitable water body, then it deposits a clutch of eggs to add to the subadult mosquitoes of various stages already present in that water body [1].

Once an adult mosquito takes an infectious bloodmeal and becomes infected, the parasite advancement beyond the midgut and into the salivary glands as infectious sporozoites requires 111 degree-days above 18˚ C [20]. Because each individual mosquito tracks degree-days since infection, infective status depends on a simple comparison of this value to 111. If the mosquito reaches this point, it becomes capable of malaria transmission to humans during subsequent blood meals. Once it has become infectious with sporozoites in the salivary glands, all subsequent bites from the mosquito are capable of transmitting malaria [1].

### *HUMAN AGENTS*

The model simulates immobile human agents, representing village inhabitants. Human agents are assumed immobile. While villagers are obviously in reality quite mobile, this may be a reasonable assumption because the mosquitoes actively seek blood-meal hosts at night [21] when villagers are sleeping in their houses. Each model grid cell marked as inhabited (digitized houses from satellite image) contains a finite number of human agents. Unless house survey data allows actual assignment of inhabitant numbers to each house, we assume a constant ten inhabitants per 10 m x 10 m village grid cell. When a mosquito enters a house to seek a bloodmeal, a certain portion of the inhabitants can be assumed protected by bednets, and if that bednet-protected host is targeted by the mosquito for a bloodmeal it will

result in the mosquito's death. If desired, repellent effects can also be included through a simple modification [1].

References

1. Bomblies A, Duchemin JB, Eltahir EAB: **Hydrology of malaria: Model development and application to a Sahelian village.** *Water Resour Res* 2008, **44**.

2. Gianotti RL, Bomblies A, Eltahir EAB: **Hydrologic modeling to screen potential environmental management methods for malaria vector control in Niger.** *Water Resour Res* 2009, **45**(8):W08438.

3. Bomblies A, Duchemin JB, Eltahir EAB: **A mechanistic approach for accurate simulation of village-scale malaria transmission.** *Malaria Journal* 2009, **8**(1):223.

4. Yamana TK, Eltahir EA: **Early warnings of the potential for malaria transmission in rural Africa using the hydrology, entomology and malaria transmission simulator (HYDREMATS).** *Malar J* 2010, **9**:323.

5. Bomblies A, Eltahir EAB: **Assessment of the impact of climate shifts on malaria transmission in the Sahel.** *EcoHealth* 2010, **6**(3):426-437.

6. Yamana TK, Eltahir EAB: **On the use of satellite-based estimates of rainfall temporal distribution to simulate the potential for malaria transmission in rural Africa.** *Water Resour Res* 2011, **47**(2):W02540.

7. Bomblies A: **Modeling the role of rainfall patterns in seasonal malaria transmission.** *Clim Change* 2012, **112**(3-4):673-685.

8. Menach AL, McKenzie FE, Flahault A, Smith DL: **The unexpected importance of mosquito oviposition behaviour for malaria: non-productive larval habitats can be sources for malaria transmission.** *Malaria Journal* 2005, **4**(1):23.

9. Minakawa N, Seda P, Yan G: **Influence of host and larval habitat distribution on the abundance of African malaria vectors in western Kenya.** *Am J Trop Med Hyg* 2002, **67**(1):32-38.

10. Charlwood J, Kihonda J, Sama S, Billingsley P, Hadji H, Verhave J, Lyimo E, Luttikhuizen P, Smith T: **The rise and fall of Anopheles arabiensis (Diptera: Culicidae) in a Tanzanian village.** *Bull Entomol Res* 1995, **85**(1):37-44.

11. Lal AMW: **Performance comparison of overland flow algorithms.** *J Hydraul Eng* 1998, **124**(4):342-349.

12. Toutin T, Gray L: **State-of-the-art of elevation extraction from satellite SAR data.** *ISPRS Journal of Photogrammetry and Remote Sensing* 2000, **55**(1):13-33.

13. Pollard D, Thompson SL: **Use of a land-surface-transfer scheme (LSX) in a global climate model: the response to doubling stomatal resistance.** *Global Planet Change* 1995, **10**(1-4):129-161.

14. d'Herbès J, Valentin C: **Land surface conditions of the Niamey region: ecological and hydrological implications.** *Journal of Hydrology* 1997, **188**:18-42.

15. Yeh PJ, Eltahir EA: **Representation of water table dynamics in a land surface scheme. Part I: Model development.** *J Clim* 2005, **18**(12):1861-1880.

16. Depinay JM, Mbogo CM, Killeen G, Knols B, Beier J, Carlson J, Dushoff J, Billingsley P, Mwambi H, Githure J, Toure AM, McKenzie FE: **A simulation model of African Anopheles ecology and population dynamics for the analysis of malaria transmission.** *Malar J* 2004, **3**:29.

17. Minakawa N, Sonye G, Yan G: **Relationships between occurrence of Anopheles gambiae sl (Diptera: Culicidae) and size and stability of larval habitats.** *J Med Entomol* 2005, **42**(3):295-300.

18. Craig MH, Snow RW, le Sueur D: **A climate-based distribution model of malaria transmission in sub-Saharan Africa.** *Parasitology Today* 1999, **15**(3):105-111.

19. Martens WJ: **Health impacts of climate change and ozone depletion: An eco-epidemiological modelling approach.** *PhD thesis.* University of Maastricht, Dept. of Mathematics, Maastricht; 1997

20. Detinova TS: **Age-grouping methods in Diptera of medical importance with special reference to some vectors of malaria.** *Monogr Ser World Health Organ* 1962, **47**:13-191.

21. Service M: *Mosquito ecology: field sampling methods:* 2nd ed. London, U.K.: Elsevier Applied Science; 1993.
